# Smart Public Transportation Sensing: Enhancing Perception and Data Management for Efficient and Safety Operations

**DOI:** 10.3390/s23229228

**Published:** 2023-11-16

**Authors:** Tianyu Zhang, Xin Jin, Song Bai, Yuxin Peng, Ye Li, Jun Zhang

**Affiliations:** 1 Shenzhen International Graduate School, Tsinghua University, Shenzhen 518055, China; tianyu-z22@mails.tsinghua.edu.cn; 2 Hangzhou DTWave Technology Co., Ltd., Hangzhou 311100, China; song.bs@dtwave-inc.com; 3 College of Mathematics and Informatics, College of Software Engineering, South China Agricultural University, Guangzhou 510642, China; 2643090040@stu.scau.edu.cn; 4 Shenzhen Institute of Advanced Technology, Chinese Academy of Sciences, Shenzhen 518055, China; liye@szbit.cn; 5 Shenzhen Institute of Beidou Applied Technology, Shenzhen 518055, China; zhangjun@szbit.cn

**Keywords:** intelligent transportation system, spatial database, edge computing

## Abstract

The use of cloud computing, big data, IoT, and mobile applications in the public transportation industry has resulted in the generation of vast and complex data, of which the large data volume and data variety have posed several obstacles to effective data sensing and processing with high efficiency in a real-time data-driven public transportation management system. To overcome the above-mentioned challenges and to guarantee optimal data availability for data sensing and processing in public transportation perception, a public transportation sensing platform is proposed to collect, integrate, and organize diverse data from different data sources. The proposed data perception platform connects multiple data systems and some edge intelligent perception devices to enable the collection of various types of data, including traveling information of passengers and transaction data of smart cards. To enable the efficient extraction of precise and detailed traveling behavior, an efficient field-level data lineage exploration method is proposed during logical plan generation and is integrated into the FlinkSQL system seamlessly. Furthermore, a row-level fine-grained permission control mechanism is adopted to support flexible data management. With these two techniques, the proposed data management system can support efficient data processing on large amounts of data and conducts comprehensive analysis and application of business data from numerous different sources to realize the value of the data with high data safety. Through operational testing in real environments, the proposed platform has proven highly efficient and effective in managing organizational operations, data assets, data life cycle, offline development, and backend administration over a large amount of various types of public transportation traffic data.

## 1. Introduction

The information technology landscape has witnessed unprecedented shifts, stimulated by the emergence of advanced technologies like cloud computing, big data, IoT, and mobile applications, among which the most impacted is public transportation [1,2,3]. The development and deployment of real-time intelligent public transportation systems were gaining traction in many urban areas worldwide. These systems leverage various technologies, such as IoT, big data analytics, AI, cloud/edge computing, and generate real-time data insight generated by data collection, integration and processing on smart traffic infrastructure, vehicular ad hoc networks, and passengers’ perception. Smart data management with low latency has greatly improved the efficiency, safety, and overall experience of smart public transportation [4].

To further harness the potential of these kinds of public transportation system, a large-scale sensing system for public transportation is crucial for accurate and efficient data collection, integration, and processing in a big data environment [5,6,7]. A key challenge facing the public transportation sensing is how to build a unified sensing system for a large variety of data sources as well as an efficient fusion mechanism for massive data. Meeting this challenge demands the design and implementation of a big data platform capable of integrating both structured and unstructured data from various departments, such as technology, operations, and human resources.

This platform aims to dismantle information silos between organizational structures, establishing a resource sharing mechanism that spans multiple levels and departments [8]. Additionally, it needs to manage, analyze, and derive actionable insights from a variety of business data, such as vehicle information, route and station data, vehicle GPS information, and operation scheduling data.

The creation of such a system, however, encounters several technical challenges, including

- Sensing various types of data: Real-time sensing of large-scale public transportation data is a crucial footstone for a big data platform [9,10]. However, it faces a series of challenges. One of the significant challenges is ensuring the accuracy and reliability of traffic data. Sensors and devices used for data sensing must be carefully calibrated and maintained to provide precise measurements. Factors such as weather conditions, environmental interferences, and sensor malfunctions can impact the accuracy of the collected data. Another challenging task is determining the optimal placement of sensors to capture accurate traffic data. Sensors should be strategically located to cover different traffic zones, including intersections, highways, and urban areas. Ensuring sufficient coverage and capturing representative data across the entire road network is a challenge, especially in large and diverse traffic environments. To overcome the above challenges, we carefully design our system and algorithms on intelligent data sensing for several downstream public transportation tasks.
- Integration of large-scale heterogeneous data: In the real-world environment, the platform is expected to link 16 different systems, including but not limited to the vehicle CAN bus platform, intelligent maintenance system, warehouse management system [11,12], and energy efficiency management platform. These systems, currently spread across disparate network environments, need to be consolidated into a singular platform, which presents a daunting task. The challenge lies not just in storing and processing such high variety of data, but also in sharing them effectively while providing real-time services. Given the wide array of data sources and the massive volume of data being processed, it is crucial for the platform to effectively track the journey of each data element from its source to its final form. However, such data tracking is typically computationally costly. Hence, an efficient data lineage exploration is essential for providing clear insights, maintaining data integrity, and supporting informed decision making.
- Data management and privacy control: Data accessing with fine-grained permission control is necessary for providing not only better data management schemes but also safer data privacy protection to prevent malicious data usage [13]. However, it requires skilled expertise and complicated data management operations. Such challenges can be overcome by establishing a row-level fine-grained permission system, ensuring that access to data is controlled at the most granular level. This enables users to access only the information they are authorized to view, promoting data security and compliance.

Existing public transportation systems have designed a series of platforms [1,14,15,16] that can handle a few types of traffic data, including vehicular GPS devices, traffic sensors, etc. However, our proposed public transportation perception system is dedicated to integrating multiple data of multiple types from multiple sources. Especially, our proposed system is designed for efficient data processing and traffic pattern discovery in edge/cloud devices; i.e., we construct a single-round trip for a passenger based on a pair of real-time transaction card records on edge devices. The efficiency and effectiveness of our data processing framework lie in our proposed efficient field-level data lineage module. Furthermore, we design a row-level permission control mechanism which can provide both data security and flexible data usage at the same time.

In summary, we construct a platform that enables unified storage, effective mining, and unified sharing of data. To make this possible, we make the following contributions:

- We have designed and implemented a series of technologies that can perform intelligent perception and processing of data at the edge and, at the same time, seamlessly integrate structured and unstructured data from different sources on the platform for unified storage and computing. Our system supports efficient data fusion of multiple data sources, including city-scale passengers’ origin–destination trip information and transactions of smart cards’ traveling information, among which a series of downstream intelligent public transportation applications are built.
- Efficient data lineage exploration techniques are proposed to provide transparency of data origin and transformation. In the scenario of large-scale multisourced data fusion for a public transportation system, it can efficiently trace back the data computation flows from downstream data insights by our proposed data lineage exploration algorithm.
- We provide a fine-grained row-level permission control mechanism for easy data management and better data privacy protection.
- Successful application in real-world environments: We have successfully implemented and applied these technologies in bus companies, proving their usefulness in providing daily operational support.

The rest of the paper is organized as follows: After a brief introduction of the related work in Section 2, we give an overview of the system, including architecture and data flow in Section 3. In Section 4, we focus on system construction introduction and difficult problem solving. Finally, we evaluate the system performance in Section 5 (visualization and human feedback) and Section 6 (quantitative experiments) and end the paper with a conclusion in Section 7.

## 2. Related Work

### 2.1. Data Platform Technologies

The implementation of big data architecture and platforms in the Internet of Vehicles (IoVs) is crucial to ensure the efficient and secure management of operations in this complex system, which integrates multiple technologies and systems. The big data architecture in IoV consists of six layers, including data acquisition, data transformation and normalization, data storage, real-time and batch data processing, data analysis, and decision making. The data acquisition layer gathers data from various sensory and observer units, which are then transformed and normalized to handle data transformation issues. Data storage manages heterogeneous data, while real-time processing is used to preprocess live data and reduce the processing load. Batch processing, employing artificial intelligence, is used for complex processes. The analysis layer performs analysis on data gathered from the real world, facilitating operational management through vehicular cloud computing techniques and big data analytics. In general, data processing in IoV can be classified into two categories: stream and batch data processing.

- Data integration: A traffic data integration platform is a system designed to integrate data from various sources related to traffic, such as sensors, GPS devices, traffic cameras, and other sources [14,15]. These platforms enable organizations to gather and integrate data related to traffic patterns, vehicle movements, and congestion levels, among other things. Traffic data integration platforms provide a range of capabilities, such as real-time data streaming, data processing, and data transformation. They are used to integrate data from various sources, such as traffic sensors, GPS devices, and traffic cameras, and provide a comprehensive view of traffic patterns and congestion levels. One of the key benefits of a traffic data integration platform is the ability to provide real-time insights into traffic patterns, enabling organizations to make better decisions related to traffic management and control. By integrating data from various sources, traffic data integration platforms can identify traffic patterns, congestion levels, and other factors that impact traffic flow, which can be used to optimize traffic management and control. There are various traffic data integration platforms available in the market, both open source and commercial. Examples of traffic data integration platforms include HERE Technologies, TomTom Traffic, and Google Maps. These platforms provide a range of capabilities and can be used by transportation and logistics companies, government agencies, and other organizations to enable real-time traffic management and control [1,14,15,16].
- Data analysis: A traffic data analysis system is a system designed to analyze traffic-related data, such as traffic patterns, congestion levels, and vehicle movements. These systems typically use a range of technologies, including big data platforms, such as Hadoop, Spark, and Flink, as well as sensory systems, such as CCD. Hadoop is a big data platform that is commonly used in traffic data analysis systems for batch processing of large volumes of data. It provides a range of tools for data storage, processing, and analysis, including HDFS (Hadoop Distributed File System) and MapReduce. Spark is another popular big data platform used in traffic data analysis systems, providing real-time data processing capabilities [2,3]. It is commonly used for streaming data processing and machine learning applications. Flink is a distributed stream processing framework used in traffic data analysis systems for real-time data processing [17,18]. It provides a range of tools for data streaming, processing, and analysis, making it a popular choice for real-time traffic data analysis. Hive is a data warehouse system built on top of Hadoop, providing SQL-like querying capabilities for large volumes of data. It is commonly used in traffic data analysis systems for ad hoc querying and analysis. Advanced Driver Assistance Systems (ADAS) [6,19] are another technology used in traffic data analysis systems, providing a range of features, such as lane departure warnings, automatic emergency braking, and adaptive cruise control. These systems use various sensors, such as radar and cameras, to gather data about the vehicle and its surroundings, which are then analyzed to provide insights into traffic patterns and other factors. Charged coupled device (CCD) sensory systems are also used in traffic data analysis systems, providing data about traffic flow, vehicle movements, and congestion levels. These sensors can be used in conjunction with big data platforms and other technologies to provide real-time insights into traffic patterns and congestion levels, enabling better traffic management and control.

### 2.2. Data Processing and Analysis Technologies

In the context of big data sensing systems for public transportation, data lineage techniques and row-level database permission controls play essential roles in managing and safeguarding data assets. Data lineage provides transparency and accountability by tracking the origin, transformations, and flow of data within the transportation system. This enables transportation authorities to gain insights into data dependencies, make informed decisions, and ensure data quality and compliance. Additionally, row-level database permission controls offer a robust security mechanism, allowing authorized individuals or entities to access and modify specific data records within the system. By leveraging these techniques, public transportation systems can enhance data governance, maintain data integrity, and strengthen overall data security in their operations.

- Data lineage techniques: Research on data lineage techniques has been the subject of several noteworthy works. In [20], the authors focused on lineage-driven fault localization, proposing techniques to identify and locate faults in data-intensive systems based on lineage information. In [21], the authors surveyed graph-based data lineage, highlighting the use of graph structures to represent and analyze lineage relationships. In [22], the authors examined big data provenance, addressing the unique challenges and opportunities associated with lineage in large-scale datasets. In [23], the authors introduced a lineage-based recomputation approach for optimizing iterative graph processing. In [24], the authors focused on automatic lineage inference for big data flows, developing techniques to automatically infer lineage relationships in large-scale data processing environments. These research works contribute to advancing data lineage techniques and their application in various domains.
- Row-level fine-grained permission control techniques: Row-level permission control techniques have been extensively studied in the field of data security and privacy. There are comprehensive surveys on fine-grained access control for both relational databases [25] and NoSQL databases [26], covering various access control models and mechanisms. A secure data access control frame that utilizes fine-grained encryption in cloud storage systems was proposed in [27]. In [28,29], the authors investigated privacy-preserving techniques for distributed data mining, enabling efficient and secure analysis on horizontally partitioned data. Furthermore, in [30], the authors presented a privacy-preserving row-level access control mechanism for healthcare data. These research works collectively contribute to the advancement of row-level permission control techniques by exploring different methodologies, such as access control models, cryptographic techniques, privacy-preserving algorithms, and blockchain-based solutions, enabling organizations to enforce fine-grained access control and protect sensitive data at the row level.

### 2.3. Smart Public Transportation Systems

Smart public transportation systems also show great potential in low-cost city-scale sensing during the process of carrying passengers [11,12]. For example, some researchers proposed to install air pollution sensors [9,10] on ride-sharing vehicles for fine-grained data collection and field reconstruction [13,31] when they move around the city. The collected data can be used to understand detailed city status, such as dark areas [32,33] and air pollution in the city corner [34,35], with the help of various reconstruction methods for better city management [36,37]. Other researchers focused on designing incentive mechanisms for such sensing and transportation dual tasks [38,39]. In addition, such ground transportation systems can also collaborate with unmanned aerial vehicles, such as indoor searching drones [40,41] and outdoor delivery drones [42,43], for a 3D sensing and transportation dual task [44,45]. Ref. [46] investigated personal mobility on autonomous vehicles under the scenario of metaverse, in which an efficient hybrid decision-making model based on OPA and RAFSI methodology under q-ROFSs is developed to evaluate the personal mobility alternative implementation options of autonomous vehicles in the metaverse. By utilizing a teaching–learning-based marine predator algorithm on the selected features, Ref. [47] proposed a novel deep-learning-based method to identify disseminating false data, which can resolve position falsification assaults in a VANET scenario when some vehicles have been hijacked and are producing false and harmful information.

## 3. System Overview

During the construction of the sensing platform, we successfully accomplished the meticulous collection and meticulous aggregation of data from diverse sources. Furthermore, we effectively utilized cutting-edge visualization and configuration tools to conduct robust big data development and processing. These endeavors yielded remarkable results, enabling us to proficiently complete the crucial tasks of data collection, aggregation, and processing. We formed a data asset system around business levels, such as people, vehicles, roads, stations, and fields. Through data applications, we provide business applications for public transportation scenarios, continuously empowering the company’s operational business.

**Figure 1:**
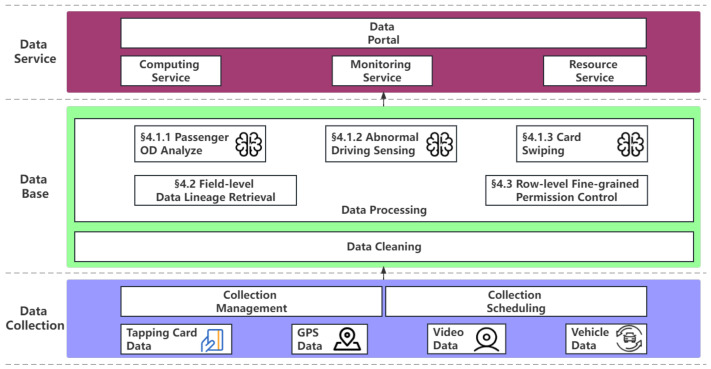
Platform functional architecture design, in which the database layer is responsible for efficient data integration, such as passenger OD collections, and its efficiency and effectiveness are supported by our proposed methods, including field-level data lineage retrieval and row-level fine-grained permission control mechanism.

The functional architecture of the platform is shown in Figure 1.

The functional architecture of the system is divided into three layers from the bottom to the top: data collection, database, and data service.

1. Data Collection

The system supports data collection in many different formats, including structured data collection, unstructured data collection, semistructured data collection, and intelligent sensing device data collection. At the same time, the system supports collection management and collection scheduling.

2. Database

The database layer mainly includes data cleaning and data processing. Data cleaning is mainly responsible for cleaning the data collected by ordinary sensors and smart sensors, including consistency check, integrity check, correctness check, formatting, and other operations. Data processing is mainly responsible for field-level data lineage retrieval and row-level fine-grained permission control. It is also responsible for data-mining-related work.

3. Data service layer

The data portal provides a unified access interface, data display, development, operation and maintenance, project management, and other function entrances.

Our architecture stands out for two main reasons. First, it incorporates data lineage techniques within the data integration and data management modules, allowing for seamless tracking of data sources even after complex integration processes. This ensures transparency and accountability throughout the data life cycle.

Second, our architecture specifically supports row-level permission data management in both the data management and project management modules. This feature empowers database managers to efficiently handle data while upholding stringent data privacy protections. By offering granular control over access permissions at the individual row level, it enhances data security and guarantees the confidentiality of sensitive information.

## 4. System Introduction

Our proposed public transportation big data system can provide fine-grained, accurate, and efficient traffic data management, which is supported by three insightful technical contributions.

- A holistic smart sensing system aiming at collections of multiple public transportation traffic data from different sources. The scope of the collected data is covering but not limited to spatiotemporal trajectories, traveling behaviors of passengers, driving behaviors of drivers, etc. We pipeline multiple series of data preprocessing, data analysis on top of such variety of traffic data, resulting in a large number of innovative intelligent public transportation traffic data applications.
- To support an efficient and accurate public transportation traffic sensing system, we design an insightful traffic data management system accounting for a massive amount of traffic big data with a high variety of data sources. To fulfill the requirements of real-time big traffic applications, the core technical challenges are tackled by our proposed public transportation traffic data management system with two technical highlights. Typically, the wide range of public transportation intelligent traffic application consists of tedious data processing and analysis phrases, in which data lineage exploration is an important operation in terms of data insight detection. We develop a data lineage exploration method that can support efficient and accurate data lineage information retrieval in FlinkSQL storage. The efficiency of our data lineage exploration method can provide highly efficient lineage exploration, which only takes less than 45 s for 200+ tables with 1000+ rows and 50+ fields (columns). Another critical technical contribution lies in the fine-grained row-level permission control in our proposed system. The row-level permission control scheme we proposed is achieved by a modifying SQL parser with additional user-oriented permission information in meta storage. Our design provides a finer grain of permission control (from table level to row level), which can help data administrators escape from splitting the same tables into multiple copies, reducing the risks of data privacy leakage.

### 4.1. Smart Sensing

The smart sensing device is mainly responsible for the perception and acquisition of data. We only illustrate the innovations of the system.

#### 4.1.1. Passenger OD Collection Equipment

**Figure 2:**
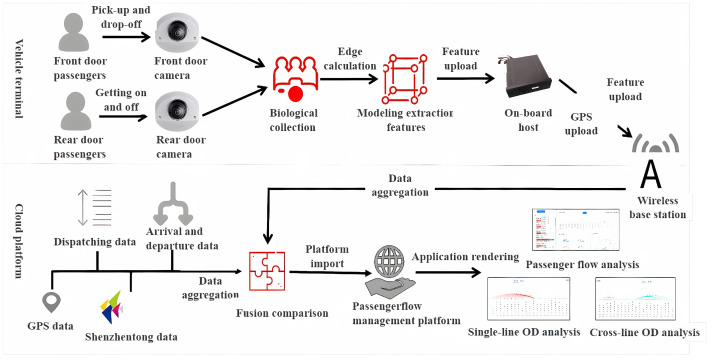
Processing flow of passenger OD collection equipment on multiple data sources.

At present, most buses use a single card swiping billing method, and it is difficult to obtain passenger OD from the ticket card data alone. Passenger OD holds great significance in optimizing vehicle structure, route direction, dispatching methods, and transport capacity allocation. The traditional OD calculation method has the problem of poor accuracy and difficulty in real time. This system deploys a set of passenger OD intelligent perception equipment on the premise of removing user privacy. The main steps are shown in Figure 2.

To optimize the utilization of passenger OD data, we have implemented a system that deploys cameras on the front and rear doors of vehicles. These cameras capture images of passengers boarding and alighting from the vehicle. However, to ensure the privacy and security of passengers, we prioritize the protection of sensitive image data. Therefore, at the edge of the network, we preprocess the images to remove any identifying information, effectively desensitizing the data. Once the images have been desensitized, we securely upload the data to the cloud for further analysis. In the cloud, we utilize ReID (re-identification) matching techniques to extract model features from the desensitized images. By comparing these features, we can accurately match and identify passengers across different boarding and alighting instances. With the matched passenger data, we then proceed to analyze and generate passenger OD results. This allows us to determine the origin and destination patterns of passengers, which serve as valuable insights for optimizing various aspects of the transportation system. By leveraging this technology, we can optimize the vehicle structure by understanding the passenger demand at different locations. This knowledge helps us determine the appropriate vehicle size and capacity required for each route. Additionally, we can optimize route directions by identifying the most efficient paths based on the passenger OD information. Moreover, these data aid in optimizing dispatching methods, allowing for more effective scheduling and reducing waiting times. Lastly, the passenger OD results assist in the optimal allocation of transport capacity, ensuring an efficient and smooth transportation experience for passengers.

#### 4.1.2. Abnormal Driving Sensing Equipment

In order to reduce public transportation safety accidents and ensure the safety of road users, we have designed and developed an intelligent sensing equipment for abnormal driving behavior. It is mainly used to detect and identify whether the vehicle is polite to pedestrians and slows down on the zebra crossing. The system mainly uses camera video data and vehicle CAN bus data as input, and judges whether the vehicle is safe to drive by identifying zebra crossings, pedestrians, and vehicle speed, and reports the identification results to the safety center platform.

In terms of courteous pedestrian detection, the intelligent perception device initially recognizes the presence of a zebra crossing and pedestrians on the crossing using camera data. Once it detects the presence of a zebra crossing ahead of the vehicle and pedestrians on the crossing, with a minimum distance of 10 m, it retrieves CAN bus data to assess whether the vehicle is decelerating. If, when the vehicle is 5 m away from the zebra crossing, the vehicle speed fails to decrease to 0 within 2 s, it is determined that the vehicle is not exhibiting courtesy towards pedestrians. The perception outcome is then transmitted to the cloud security platform as an alarm. The recognition accuracy rate is more than 95% during the day and more than 90% at night.

When it comes to speeding at the zebra crossing, the intelligent perception device utilizes camera data to identify both the zebra crossing and pedestrians. If the zebra crossing is recognized but no pedestrians are detected, the process of zebra crossing speeding detection is activated. The system retrieves CAN bus data to estimate the vehicle’s speed. If the vehicle remains above 20 km/h for more than 5 s and is within 10 m of the zebra crossing, it is deemed to be speeding on the zebra crossing. The system promptly transmits the detection result to the cloud security platform as an alert. The accuracy of recognition is more than 95% during daylight and more than 90% at night.

#### 4.1.3. Card Swiping Equipment

Traditional card swiping devices can only be charged according to the billing rules set in advance. However, due to the secondment of vehicles, the charging rules of different lines are different. At this time, it becomes very difficult to modify the billing rules of card swiping devices. This system is transformed on the basis of the original card swiping equipment, so that it can be interconnected with intelligent dispatching equipment; automatically synchronize lines, stations, and billing information; and automatically modify billing policies. The overall process is as follows.

- Modify the vehicle-mounted card reader software, regularly receive the vehicle location information synchronized by the intelligent dispatching device, and perform logical processing before saving, such as filtering abnormal sites and abnormal directions.
- When the passenger swipes the card or scans the code, the fee will be deducted according to the normal transaction process, and the current line number, vehicle number, driving direction, station number, and other information of the current card reader will be saved in the transaction record.
- In the IC card transaction process, it will be judged that the ’lock flag’ in the ’transit segment application’ in the card is 01; then the fare replenishment process will be carried out, and the fare will be deducted according to the ’maximum deduction amount’, and then the normal operation will be performed again. The one-ticket transaction process completes the deduction. Otherwise, complete the deduction according to the normal one-ticket transaction process.
- Through the 4G wireless network of the on-board card reader, upload the card swiping/code scanning transaction to the enterprise platform system.

Furthermore, we have introduced encryption techniques, an authorization verification mechanism, real-time monitoring, and abnormality detection techniques to ensure the safe card swiping transaction.

### 4.2. Efficient Data Lineage Exploration

Data lineage queries refer to the process of tracking and analyzing the history of data sources, processing, and usage in data management systems. For a spatiotemporal traffic database, data lineage queries can help understand the source of traffic data, how it was collected, processed, and used, thereby improving the quality, reliability, and usability of the data. In our data sensing system, these features can enable efficient data tracing back to abnormality in original data sources or data processing when events are detected in downstream applications, including passenger flow analysis, single-line origin–destination analysis, etc.

#### 4.2.1. Proposed Technique—Efficient FlinkSQL Field-Level Data Lineage Retrieval

Data lineage is a method of describing the source, destination, and transformation processes of data throughout its life cycle. It can help organizations understand and manage their data assets to better utilize them. In our system, we propose a technique to explore field-level data lineage in FlinkSQL. In the current implementation, there are two major methods to recover data lineage relationship: the first one is analyzing the physical execution plan generated by a FlinkSQL engine to retrieve field-level data lineage, and the other one is analyzing the whole set of SQL statements to retrieve the data lineage of FlinkSQL. However, we have encountered challenges while utilizing these existing techniques into our environment.

The main workflow of analyzing data lineage based on a physical execution plan is to recover the data relationship from the StreamGraph generated alongside the physical execution plan. This process faces two major obstacles. On one hand, the process of generating the execution plan for data lineage analysis is costly in computation. On the other hand, the result of the analysis based on the physical execution plan is inaccurate due to the fact that the data lineage can be lost for many-to-one field mapping as well as UDTF-supported fields in the physical execution plan.

Data lineage can also be identified by utilizing a customized SQL statement analyzer on SQL statements, and it cannot achieve desirable performance due to similar reasons: the first one is that the time complexity is extremely high for the SQL statement analyzer module since it has to take all FlinkSQL-supported SQL functions into account in order to generate a data lineage with no error; the second one is that it comes with very low extensibility in that the SQL statement analyzer module has to be updated whenever a new type of SQL statement is additionally supported by FlinkSQL.

To overcome the above challenges and to provide a data lineage retrieval service with both high efficiency and accuracy, we design a data lineage method based on the logical plan generated at the early stage of SQL query processing in FlinkSQL. Our method can take advantage of two aspects:

- We derive the data lineage relationship in the field level from the logical plan generated at the early stage in query planning, escaping the costly generation of a physical execution plan. This enables high efficiency of our retrieval on data lineage.
- The exact data lineage relationship can be extracted from relational metadata accessing APIs provided by Calcite, which is the core SQL planning and optimizing part in FlinkSQL, which can guarantee that the identified data lineage is correct, resulting our overall processing with 100% accuracy. Furthermore, a self-defined SQL function is supported, which can be easily extended to allow the latest features of FlinkSQL to be implemented into our framework.

#### 4.2.2. Algorithm

**Figure 3:**
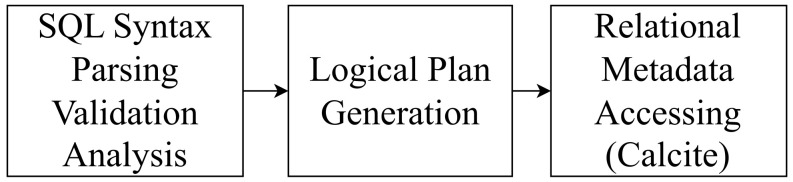
Overview of our data lineage retrieval algorithm.

In a nutshell, our proposed algorithm of field-level data lineage retrieval for FlinkSQL can be highlighted by the following three steps as demonstrated in Figure 3:

1. In the first step, a RelNode tree is generated by the SQL parser, validator, and analyzer of a FlinkSQL engine, which represents the building blocks of the query execution plan, including data sources, data operations, and their processing flows.
2. Afterwards, the optimization processing is conducted on the original RelNode tree to generate an optimized logical execution plan for the SQL query. In our proposed algorithm, an optimized RelNode tree is produced by the logical planning only, rather than the physical execution planning.
3. In the last step, we utilize the inner function RelMetadataQuery.getColumnOrigins() in Apache Calcite to retrieve the metadata of the optimized RelNode tree nodes efficiently, based on which we establish the overall data lineage relationship information data structure.

The detailed workflow of our proposed algorithm is demonstrated in Algorithm  1. The input of the overall algorithm is an SQL statement for data processing, and it generates the field-level data lineage relationship information. In Line 1, a whole processing on the input SQL statement is conducted by the SQL syntax processing engine inside FlinkSQL. The original RelNode tree is produced after three main procedures: (a) Syntax parsing (Parse) involves using JavaCC to convert input SQL into an abstract syntax tree (AST), which is represented in Calcite by the SqlNode type. (b) Syntax validation (Validate) involves validating the syntax based on metadata information, such as whether the queried tables, fields, and functions exist. The validation is carried out for each clause, such as FROM, WHERE, GROUP BY, HAVING, SELECT, and ORDER BY, and the validated result is still an AST represented by the SqlNode type. (c) Semantic analysis (Convert) involves constructing a relational expression (RelNode) tree based on the SqlNode type AST and metadata information. This RelNode tree represents the original logical plan (original RelNode tree).
**Algorithm 1:** Overview of field-level data lineage retrieval algorithm**Data**: $SQL_{input}$: An SQL statement of data processing**Result**: $LINEAGE_{info}$: Field-level data lineage relationship info data structure$RelNode\_Tree_{origin}\leftarrow$ SQL_SYNTAX_PROCESSOR($SQL_{input}$)$RelNode\_Tree_{opt}\leftarrow$ LOGICAL_PLAN_OPT($RelNode\_Tree_{origin}$)$LINEAGE_{info}\leftarrow$ GET_COLUMN_ORIGINS($RelNode\_Tree_{opt}$)

In the optimization phase, FlinkSQL originally had 12 steps, namely, subquery_rewrite, temporal_join_rewrite, decorrelate, default_rewrite, predicate_pushdown, join_reorder, project_rewrite, logical, logical_rewrite, time_indicator, physical, and physical_rewrite.

**Figure 4:**
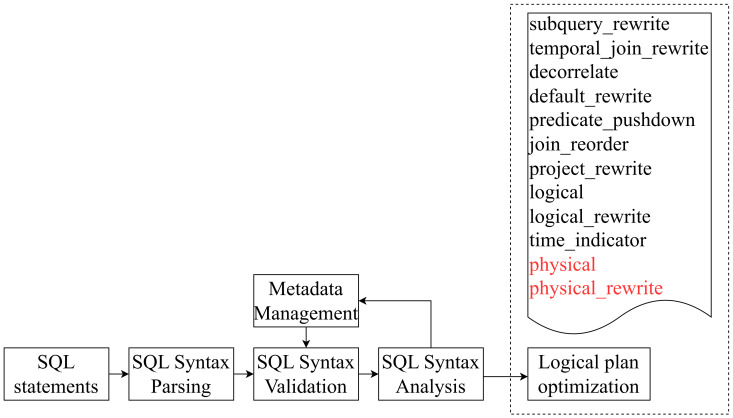
Overview of logical execution plan optimization (steps in red are to be removed).

After going through this series of optimization steps, the optimized physical plan is obtained. However, this plan cannot utilize the capabilities provided by Calcite to obtain the lineage relationship between fields. Therefore, this invention removes the last two optimization steps, physical and physical_rewrite, and only generates the optimized logical plan. This plan is also a relational expression (RelNode) tree and is called the optimized RelNode tree. The overall processing is shown in Figure 4.

The final stage mainly involves using the relational metadata access interface provided by Apache Calcite to obtain the lineage relationships, with the following steps: (a) Obtain the output table and output field list based on the AST. (b) For each output field, call the RelMetadataQuery.getColumnOrigins() method to obtain the source table and source field. This method takes two parameters, the first being the optimized logical plan (optimized RelNode tree type) generated in the previous step, and the second being the output field. Therefore, this method essentially retrieves the mapping relationship between the input and output fields from the optimized RelNode tree. Finally, the lineage mapping relationship is constructed, and the final result is returned to the user for display, as shown in Table 1. This lineage relationship represents the result of joining table A and table B and inserting it into table C, where both field 2 of table A and field 3 of table B are mapped to field 6 of table C.

After going through the above steps, it is possible to accurately obtain the lineage relationship between fields. If FlinkSQL introduces a new syntax in future versions, the lineage relationship between fields can be accurately parsed by a custom plugin. The new syntax introduced in FlinkSQL corresponds to a subtype of SqlNode, as shown in Table 2 in which the first two columns of the following table for Lookup Join, UDTF, and Watermark and the last column are the custom plugin.

### 4.3. Row-Level Fine-Grained Permission Control

Row-level permission control in relational databases refers to the ability to restrict access to individual rows of data in a database table based on the privileges granted to a user or role. This means that users can be given permission to view or modify specific rows of data while being denied access to others.

Row-level permission control is typically implemented using database security features, such as access control lists (ACLs), role-based access control (RBAC), or a combination of both. These features allow database administrators to define permissions at a granular level, specifying which users or roles can access specific rows of data within a table.

#### Proposed Techniques—Fine-Grained Row-Level Permission Control in FlinkSQL

Row-level permissions are a method of horizontal data security protection that can solve the problem of different users only being able to access different data rows. For example, in an order table, User A can only view data in the Beijing region, while User B can only view data in the Hangzhou region. Currently, with the rise of real-time data warehouses (represented by Flink), there is an urgent need for a method and device to control row-level permissions in Flink SQL.

**Figure 5:**
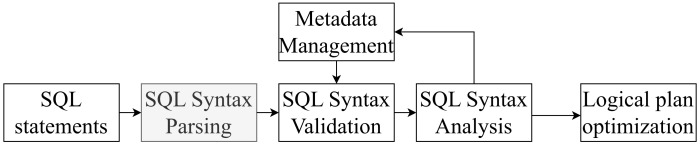
Overview of our fine-grained row-level permission control.

The execution process for FlinkSQL is demonstrated in Figure 5. After a user inputs an SQL statement, an abstract syntax tree (AST) is generated during the syntax analysis stage. The present invention generates a new AST for row-level permission control by assembling row-level filtering conditions during the parsing stage.

During the parsing stage, if the input SQL statement includes a query operation on a table, a Calcite SqlSelect object is created. To restrict row-level permissions on the table, it is only necessary to intercept the where condition when constructing the Calcite SqlSelect object, rather than parsing various SQL statements to search for tables with row-level permission constraints.

**Figure 6:**
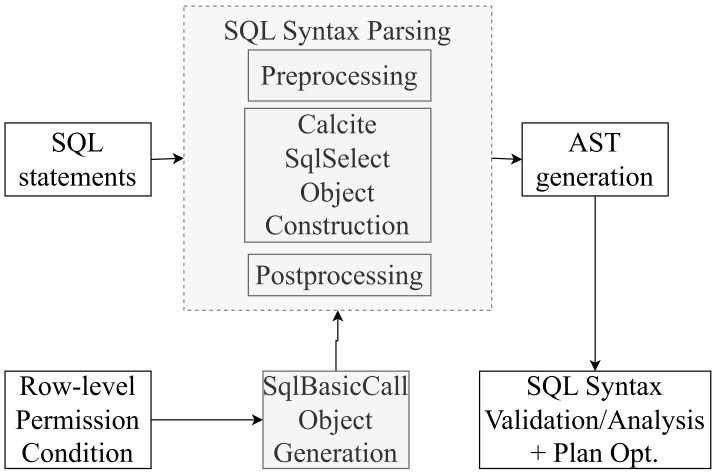
Detailed steps for row-permission control.

When constructing the where condition of the SqlSelect object, the row-level constraints are looked up based on the username and tablename of the SQL execution. Calcite is then used to parse the expression to generate a SqlBasicCall object. Next, the original where condition and the configured row-level permission condition SqlBasicCall are reassembled to generate a new where condition with row-level filtering, which is the new AST. Finally, the new AST is used to perform syntax checking, semantic analysis, optimization, and execution. The entire process is transparent and imperceptible to the user executing the SQL statement, who can still use Flink’s built-in execution methods without requiring additional configuration. The detailed steps for our proposed technique can be referred to Figure 6.

We also demonstrate a running example in Table 3. For the row-level permission condition defined, where User A can only view data for the Beijing region and User B can only view data for the Hangzhou region in the orders table:

When both User A and User B execute the same query ’SELECT * FROM orders’, the system will automatically assemble the row-level constraints onto the original SQL statement. Below are the two SQL statements that User A and User B will execute, respectively:

- User A’s SQL statement: SELECT * FROM orders WHERE region = ’Beijing’;
- User B’s SQL statement: SELECT * FROM orders WHERE region = ’Hangzhou’.

## 5. System Modules

The platform offers a comprehensive suite of modules designed to support big data development and management. These modules include the organization management, data asset, data service, offline development, and backend management modules. Together, they provide a comprehensive solution for managing and analyzing big data, enabling users to efficiently develop and manage data assets, build and deploy data services, and manage platform resources and operations.

### 5.1. Organization Management

The organization management module is a key component of the platform. This module provides organization administrators with a suite of tools to configure and manage various aspects of their projects before development begins. The module includes user management, cluster management, resource management, data source management, project management, instance management, alert notifications, and access keys. It provides administrators with a comprehensive suite of tools for managing various aspects of their projects. With this module, administrators can configure and manage projects, project environments, execution environments, data sources, resource groups, execution agent services, metric monitoring, clusters, cluster services, cluster users, and cluster roles.

The main functionality system of the organization management module is demonstrated in Table 4.

### 5.2. Data Asset

**Figure 7:**
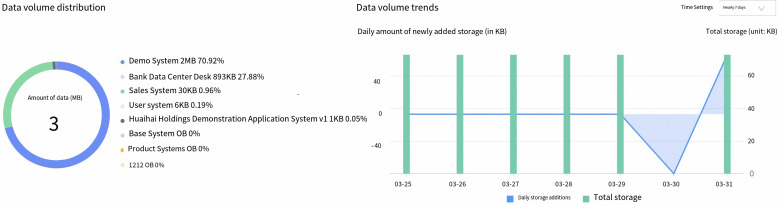
Overview of data asset module (usage monitoring and trending).

The data asset module is a comprehensive suite for managing the full life cycle of data assets. It provides panoramic view, data mapping, data standards, data models, data quality, and data security functions. Data management and users can use the panoramic view and data mapping to inventory and organize data assets. They can also use the data search service to quickly locate corresponding data. The module provides a data standard definition tool with built-in national and industry standards. Developers can use these standards for visual modeling and configure quality audits based on the standards to improve data quality. We demonstrate the data asset module in our system in Figure 7, where an overview of data usages by different components can be easily shown with monitoring on data usage trending.

### 5.3. Data Service

**Figure 8:**
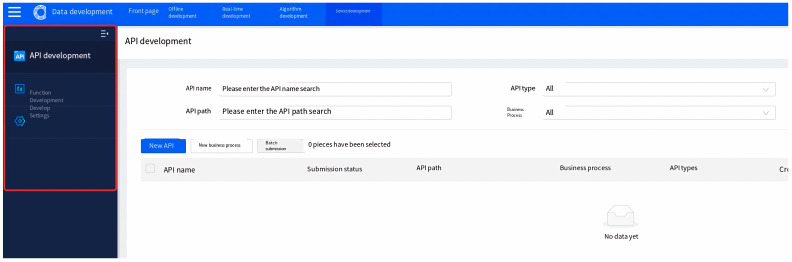
Overview of data service module. API name, path and type are configurable in data API development page.

The data service module is a comprehensive tool for managing the full life cycle of APIs, providing fine-grained, high-availability APIs for data querying, label querying, intelligent computing, and more. It supports API generation and registration in two modes, as well as one-stop API publishing, security authentication, and call flow control services, facilitating data openness and sharing. An overview of the data service module is demonstrated in Figure 8.

API (application programming interface) is a capability encapsulating data querying and processing, typically containing basic information, request paths, and protocol-related request parameters. Services are the capabilities of APIs for user access through the API gateway. Data sources are the connection information of databases, and data APIs access data through data source connection information, which needs to be added in data source management.

Algorithm models are function-computing objects generated through machine learning or deep learning, enabling complex business computing or processing. They are typically managed in algorithm development in the DataWorks platform.

A highlighted feature in the proposed system inside the algorithm model is that the computation workflow of the data processing is fully demonstrated, where multiple algorithm components are organized as a directed acyclic graph to represent their workflow.

**Figure 9:**
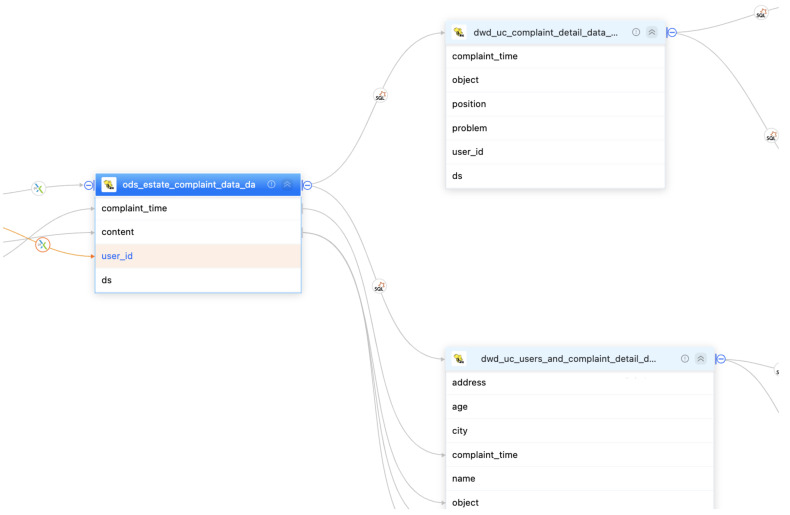
An example of data-lineage-supported module. The two tables on the right are derived from the data processing based on the left table.

Furthermore, a significant aspect of the proposed system is its support for efficient data lineage (Figure 9). The system is designed to capture and track the origin, transformations, and flow of data throughout the entire data processing life cycle. This comprehensive data lineage capability allows users to easily trace the path of data from its source to its destination, enabling transparency and accountability in data processing.

In summary, the data service module provides a comprehensive tool for managing APIs, including data querying, label querying, and intelligent computing, among others, as well as capabilities such as submission, debugging, testing, deployment, undeployment, authentication, algorithm models, algorithm experiments, third-party authentication, and functions.

### 5.4. Offline Development

The offline development module is a one-stop big data development environment provided by the platform, offering a full-chain solution for data synchronization, development, publishing management, and operational monitoring. It can be used to build PB-level data warehouses and achieve large-scale data integration, enabling the capitalization of data through deep value mining.

**Figure 10:**
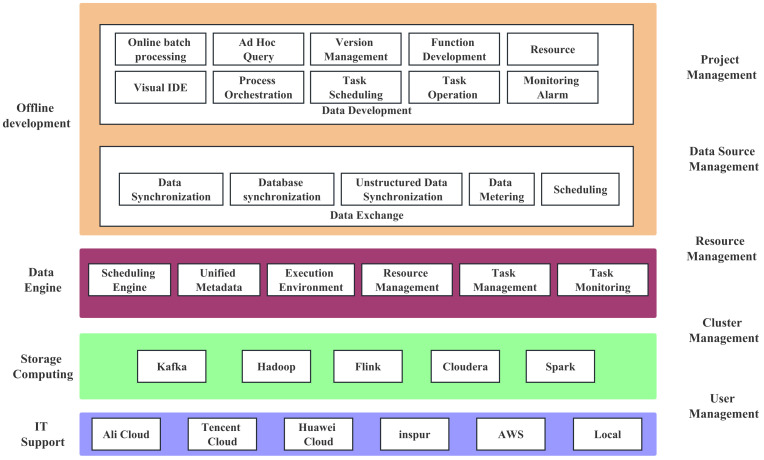
Overview of the offline development module.

The overall functional architecture is shown in Figure 10. The offline development module provides a comprehensive solution for big data development and management, enabling the construction of large-scale data warehouses and deep value mining of data assets. It supports a range of job types and functions, facilitates job version tracking, and provides operational monitoring and data reprocessing capabilities.

### 5.5. Backend Management

The backend management module is a critical component of the platform, providing a range of capabilities for managing tenants, accounts, and features, as well as configuring data source types, job types, vendors, and vendor services. Through this module, users can view license information, ensuring compliance with software usage rights and obligations. It provides a centralized platform for managing tenants, accounts, and features on the platform, as well as configuring data source types, job types, vendors, and vendor services. Through this module, users can view license information and ensure compliance with software usage rights and obligations.

## 6. Evaluation

To further evaluate the efficiency and effectiveness of our proposed system qualitatively and quantitatively, we conducted a series of data processing and management tasks on an experimental environment with six computing nodes. Each of them consists of CPU of four cores and a memory of 16 GB and 100 GB storage with a running JVM of up to 2 GB memory. We evaluated our platform from six different dimensions to ensure that it met the performance requirements. The following are the results of our testing in each dimension.

### 6.1. Efficiency

**Figure 11:**
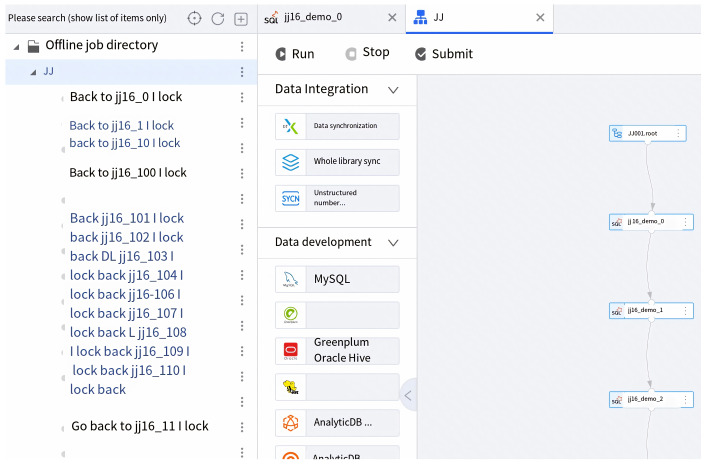
Experiment demonstration of metadata collection. Metadata relationship is revealed on the right.

**Figure 12:**
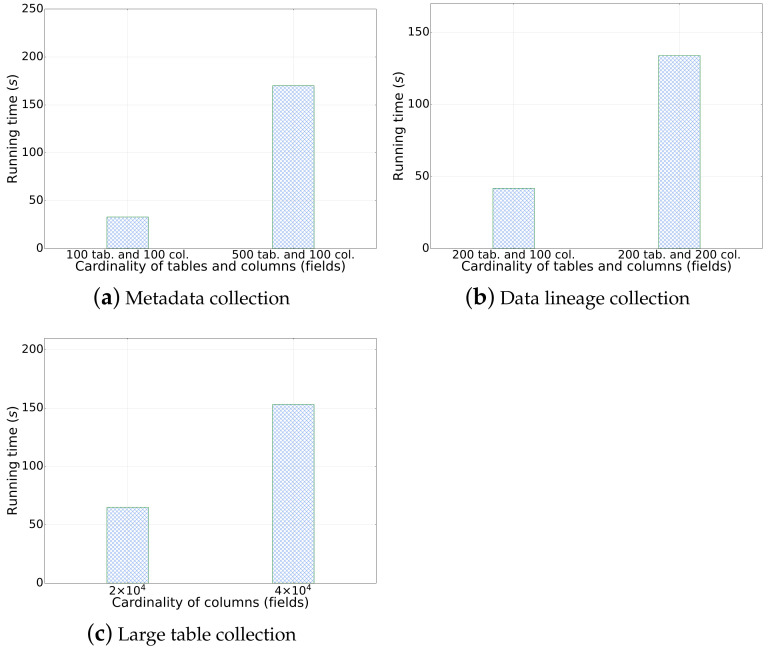
Scalability of metadata collection, data lineage collection, and large table collection.

**Figure 13:**
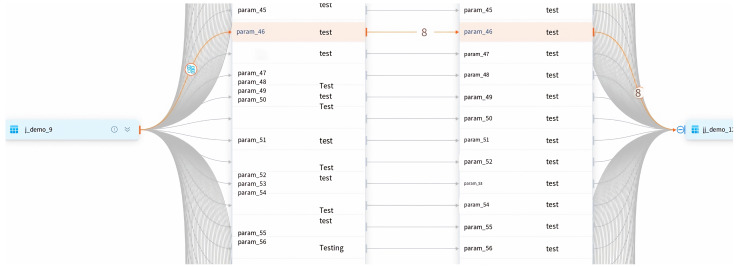
Experiment demonstration of data lineage. The columns of the right tables are computed based on the columns of the left tables.

**Figure 14:**
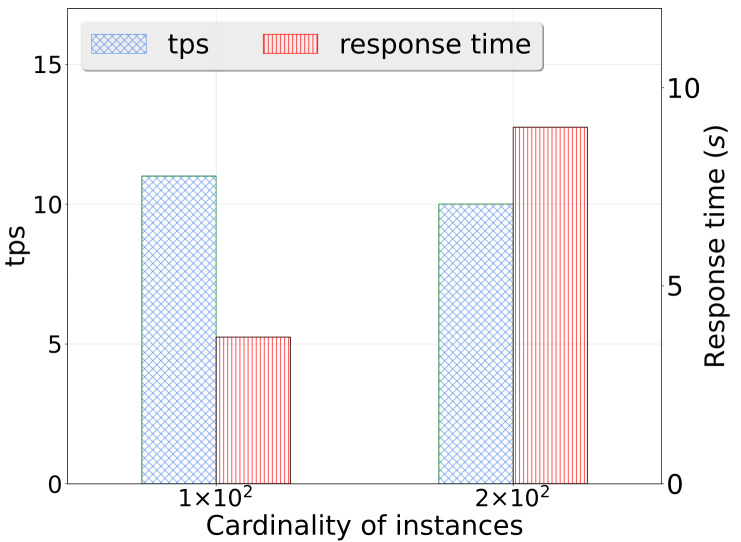
Response time of concurrent data processing.

**Figure 15:**
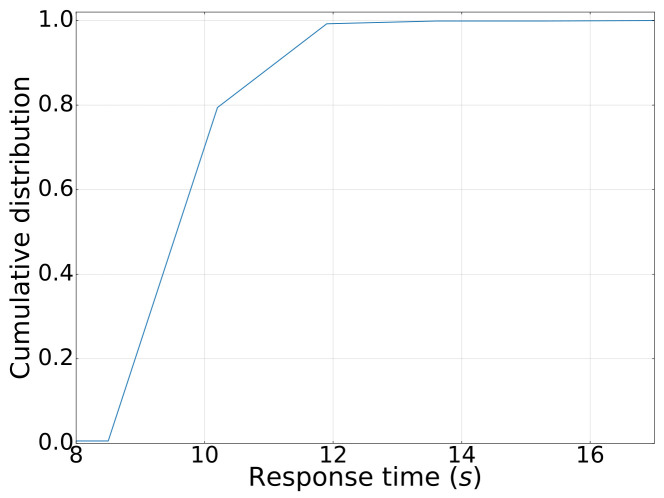
CDF of response time of concurrent data processing.

- Metadata collection: We tested the metadata collection process on two nodes using version 5.5 of our platform. The metadata relationship can be revealed by our system as in Figure 11. Through extensive experiments, our system has been demonstrated to successfully retrieve in total 20,002 records of metadata as well as the relationship with only 13.1 min, in which the average collection time is found to be satisfactory and well within the expected range. We also investigated the scalability of the metadata collection process in our system. With varying scales of tables with the same size of data fields (columns), we found that the running time of the metadata collection process is linear to the size of data collection as shown in Figure 12a.
- Data lineage collection: In this test scenario, we collected lineage data for a single table with 50+ fields, 1000+ rows, and 200 tables using SparkSQL as shown in Figure 13. The average lineage delay was found to be 45 s, which is within the acceptable range. In comparison, we conducted baseline techniques that process data lineage over StreamGraph (which is generated after the physical execution planning). We observed that the baseline technique generates an inaccurate data lineage relationship with at least two times of processing time, rendering the efficiency and effectiveness of our proposed method. Similarly, we also investigated the scalability of our data lineage collection process, and the result is demonstrated in Figure 12b. We tested our proposed method on two cases with the same 200 tables but different numbers of columns (100 and 200, respectively). For the case that only 100 columns were involved, the running time of data lineage collection was 41.83 s, compared with 133.84 s for the case with 200 columns. We observed that the computation time was increased near-linearly to the increasing on data size, which showed that our proposed method is efficient for large-scale data processing in real application.
- Row-level permission control: We conducted an evaluation of the row-level permission control module to assess its performance and effectiveness in our system. Through various tests and analysis, we found that the module successfully enforced row-level permissions, ensuring secure and controlled access to sensitive data. The performance impact was minimal, with no noticeable delays or latency issues observed compared with the baseline technique in which tables are manually separated by a DB administrator based on user authorization. Hence, it showed scalability, efficiently handling increasing data volumes and user demands.
- Large table collection: We tested the platform’s ability to collect metadata for a single table with 20,000 fields. The average collection time was found to be 108.704 s, which is within the expected range. The scalability of a large table collection was also tested on another datasets with varying fields as shown in Figure 12c. It demonstrated consistent results with our metadata collection module that our data collection module has linear-time computation cost with data cardinality.
- Scheduling delay restriction: We created 500+ workflows for offline development, each with 100+ jobs, and ran jobs to record the time difference between scheduling and job execution. The average delay time for scheduling among the 500 workflows was found to be 2 s, which is well within the acceptable range.
- Execution concurrency: We created 500+ workflows for offline development, each with 100+ jobs, and ran jobs with 100 and 200 concurrencies. The throughput per second (tps) and average response time for job execution were found to be a satisfactory level with tps of 11 and 10, response time of 3.7 s and 9.8 s, respectively (Figure 14). It meets the performance requirements of our clients. We have also demonstrated the cumulative distribution of response times of concurrent jobs in Figure 15, in which we can observe that most jobs can be finished within 10 s.

### 6.2. Effectiveness

We also verified the effectiveness of our proposed system on the sensing flow of passenger OD. We gathered on-field video data and prepared ground-truth data created by a human. Specifically, we demonstrated the experimental study on two representative applications of our system, which are listed as follows:

- Passenger counting: We collected multiple on-field video data recorded on a bus that worked on schedule. Multiple videos were collected, capturing passengers who were going on board or taking off the bus. The ground truth data were provided by a human being identifying the real number of passengers on multiple scenarios. Out of the average ground truth number of all 134 on-board passengers, our system can automatically identify on average 131 of them, which indicates that our system can have an accuracy of 97.76% in the mission of passenger counting.
- Passengers OD collection: Based on the same set of experimental video data, we also prepared ground truth passenger ODs by identifying the on/off stations of the same passenger. Out of the average 134 on-board passengers in multiple videos, our system can retrieve on average 120 pairs of correct OD pairs, which indicates that our system can on average have an accuracy of 90.30% in the mission of passenger OD collection.

In conclusion, our platform’s performance was evaluated using various scenarios, and the results met the expectations of our clients. The metadata and lineage collection, large table collection, scheduling delay restriction, and execution concurrency were all tested and found to be satisfactory in terms of efficiency. Additionally, the passenger counting and OD collection method can correctly retrieve passenger information with an accuracy of over 90%.

### 6.3. Data Availability Statement

The public transportation big traffic data serving in our proposed system is not applicable to be shared to any other entity, which is a critical request from our data provider for the reason that anonymous traveling data can still be sensitive due to privacy problem. Thus, we are not allowed to provide data accessibility to others per our data provider’s request.

## 7. Conclusions

This paper presents a novel public transportation sensing platform that effectively integrates, organizes, and analyzes complex data generated from various sources in the public transportation industry. The platform achieves the intelligent sensing of origin–destination data, abnormal driving, and card swiping. To overcome the challenges in data processing brought by data variety and data volume, the platform introduces an efficient field-level data lineage exploration method to enable effective and efficient smart data sensing, including passenger OD collection, abnormal driving sensing, etc., on a large number of traffic data. Furthermore, a fine-grained row-level permission control mechanism is introduced to ensure unified storage, effective access, and data sharing. Real-world human usage experience investigation and quantitative experiments conducted demonstrate the platform’s ease of usage in managing organizational operations, data assets, data life cycle, offline development, and backend administration. Additionally, experimental results from quantitative experiments show that our system provides effective data integration and processing (over 97% for passenger counting and over 90% for passenger OD collection). Ultimately, our system can greatly improve the overall public transportation experience with multiple intelligent applications over a large amount of various types of public transportation traffic data. At this stage, our system solely focuses on data sensing and data management submodules of a large ITS system. In the future, we will continue to investigate the integration of a large traffic AI model and an efficient public transportation perception system for better data insight discovery and a smarter intelligent traffic management.

## Figures and Tables

**Table 1:** Field mapping in data lineage example.

Source Table	Source Field	Output Table	Output Field
Table A	Field 1	Table C	Field 5
Table A	Field 2	Table C	Field 6
Table B	Field 3	Table C	Field 6
Table B	Field 4	Table C	Field 7

**Table 2:** Examples of new FlinkSQL features.

FlinkSQL New Features	Subtype of RelNode	Custom Plugin
Lookup Join	Snapshot	getColumnOrigins(Snapshot rel)
UDTF	Correlate	getColumnOrigins(Correlate rel)
Watermark	WatermarkAssigner	getColumnOrigins(WatermarkAssigner rel)

**Table 3:** Running example on fine-grained row-level permission control.

ID	User Name	Table Name	Row-Levewl Permission Condition
1	User A	orders	region = ‘Beijing’
2	User B	orders	region = ‘Hangzhou’

**Table 4:** List of submodules of the organization management module.

Submodule	Functionality
Project Management	Adding/configuring/deleting/banning projects
Data Source Management	Adding/configuring/authoring data sources
Resources Management	Adding/configuring/authoring/checking servers and their status
Clusters Management	Adding/removing/configuring/authoring clusters
Instance Management	Adding/configuring/authoring instances and database management
Alerting System	Management on alters based on defined rules
Account/User Management	Adding/configuring/authoring/deleting users and accounts

## Data Availability

The data are not publicly available due to the privacy concerned of our data provider.
